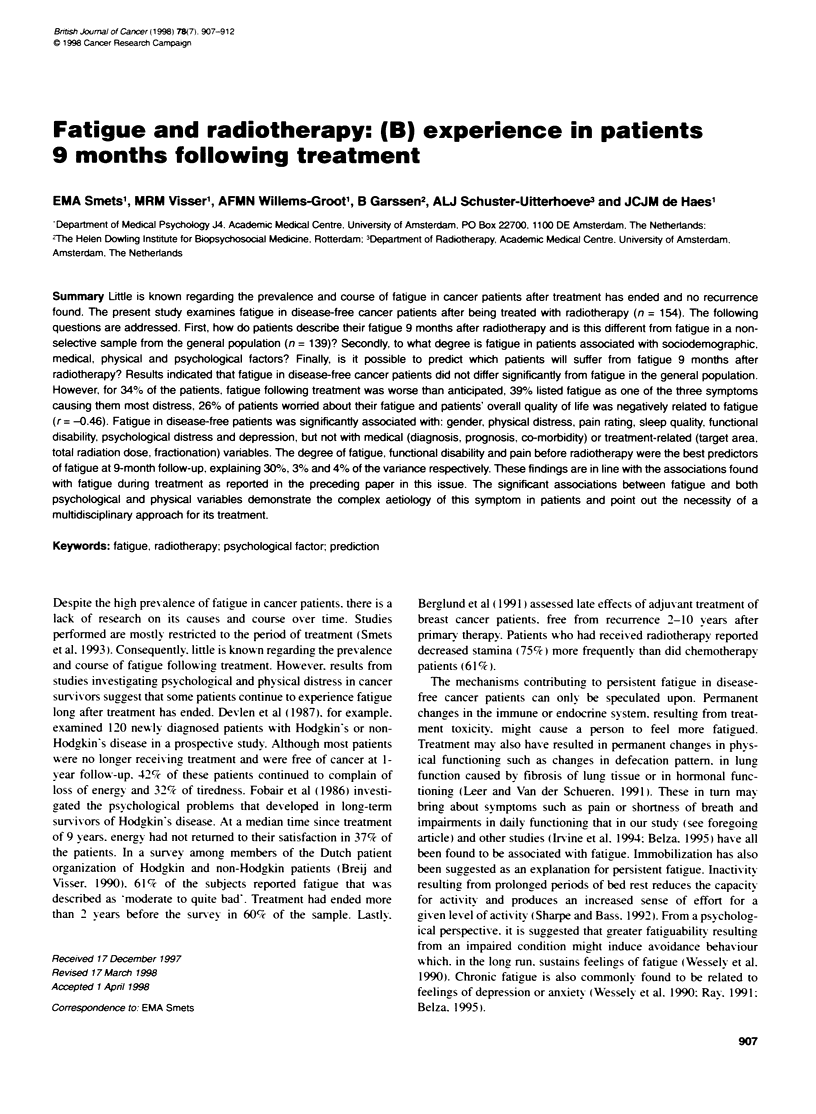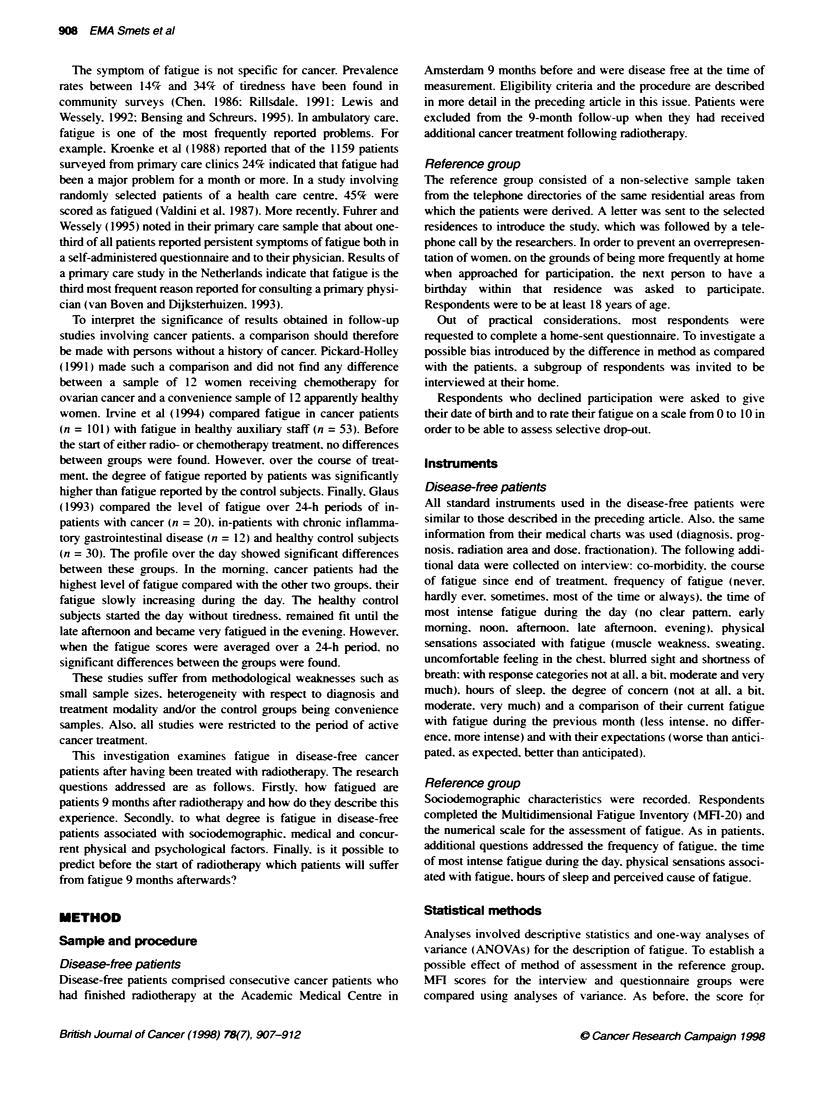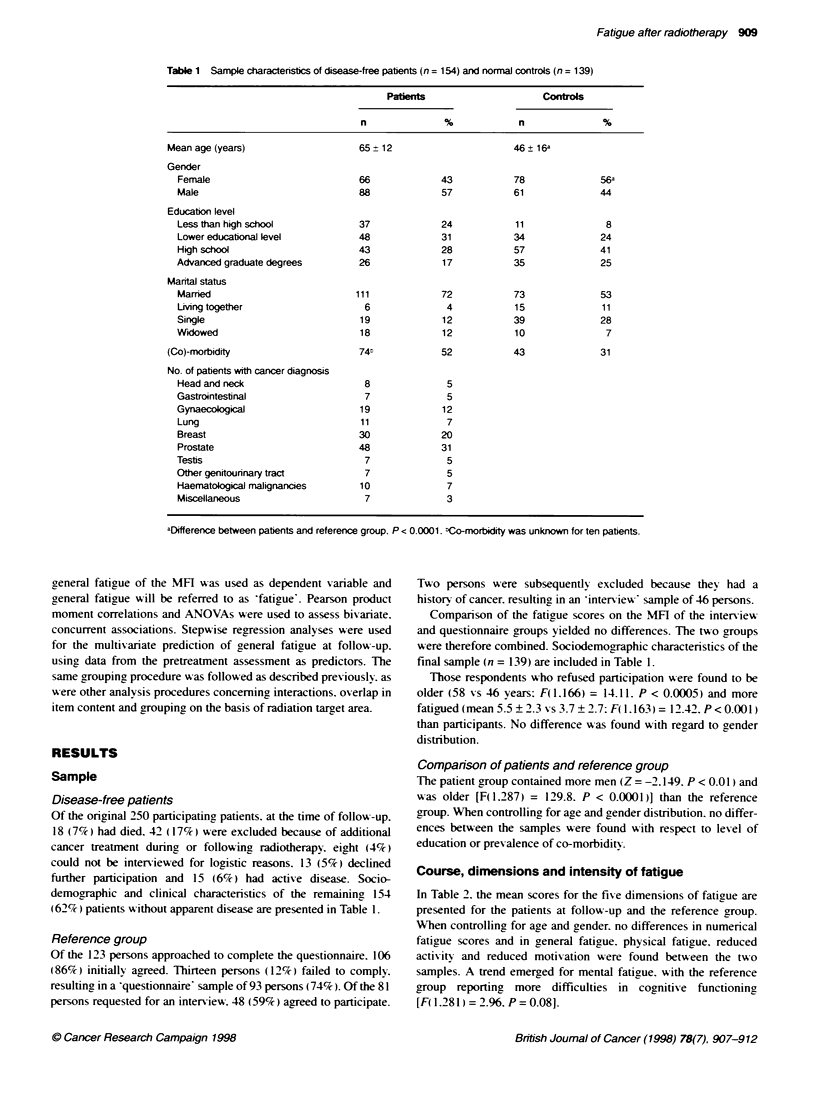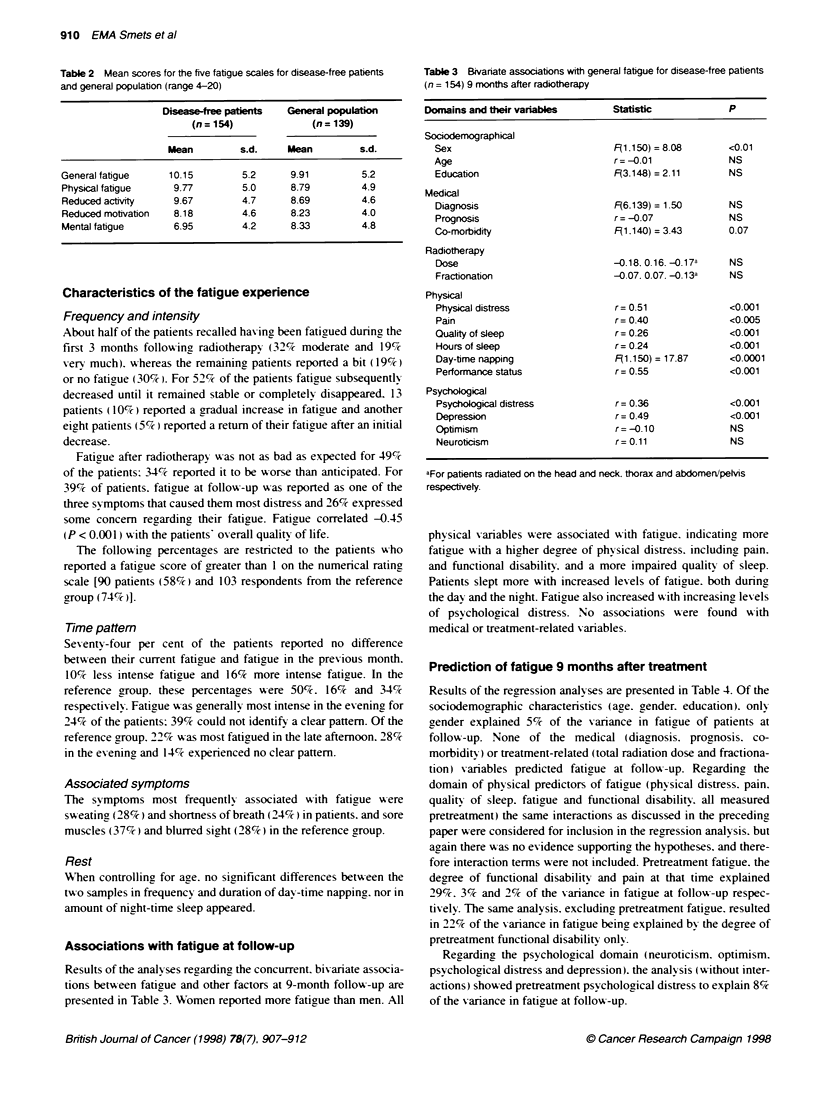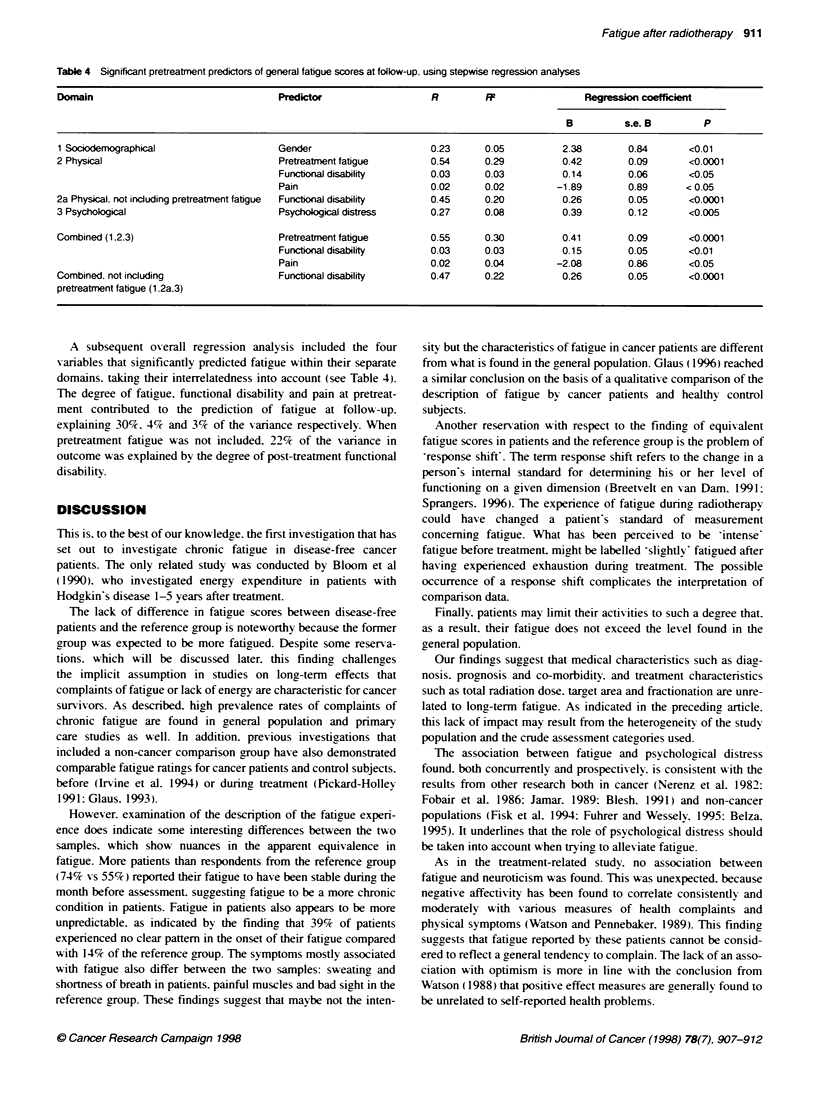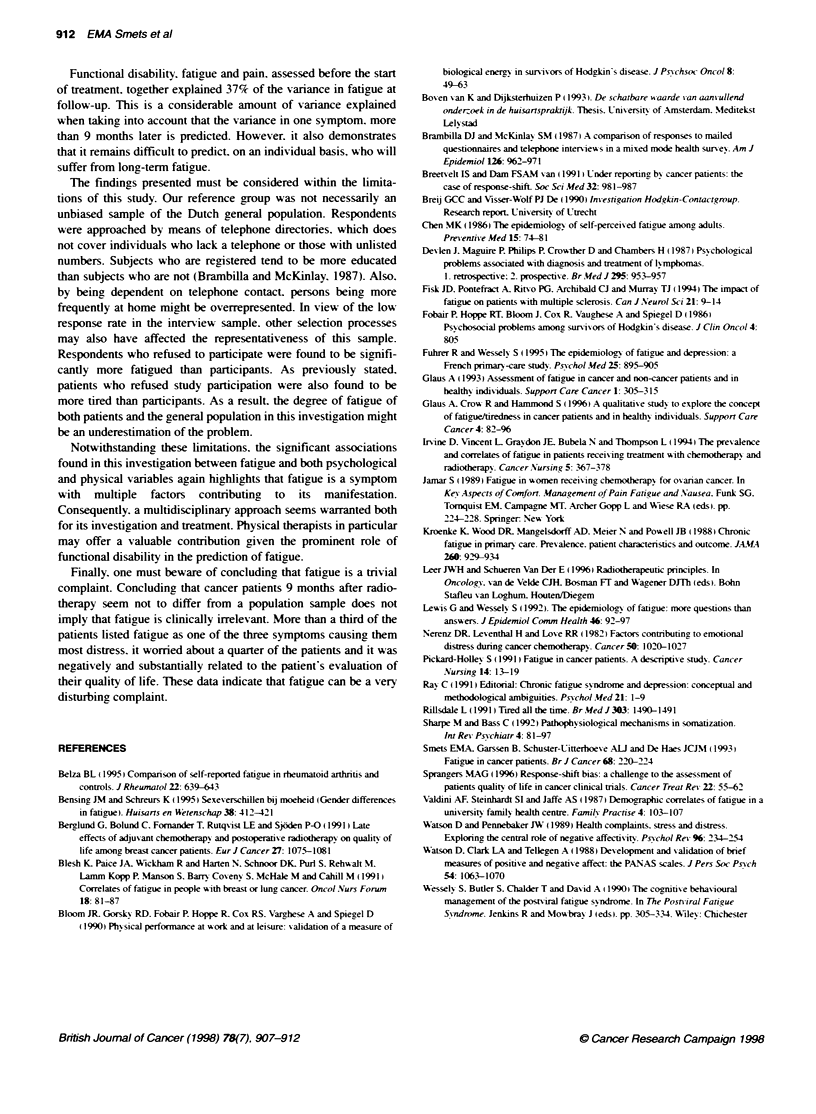# Fatigue and radiotherapy: (B) experience in patients 9 months following treatment.

**DOI:** 10.1038/bjc.1998.600

**Published:** 1998-10

**Authors:** E. M. Smets, M. R. Visser, A. F. Willems-Groot, B. Garssen, A. L. Schuster-Uitterhoeve, J. C. de Haes

**Affiliations:** Department of Medical Psychology, Academic Medical Centre, University of Amsterdam, The Netherlands.

## Abstract

Little is known regarding the prevalence and course of fatigue in cancer patients after treatment has ended and no recurrence found. The present study examines fatigue in disease-free cancer patients after being treated with radiotherapy (n = 154). The following questions are addressed. First, how do patients describe their fatigue 9 months after radiotherapy and is this different from fatigue in a nonselective sample from the general population (n = 139)? Secondly, to what degree is fatigue in patients associated with sociodemographic, medical, physical and psychological factors? Finally, is it possible to predict which patients will suffer from fatigue 9 months after radiotherapy? Results indicated that fatigue in disease-free cancer patients did not differ significantly from fatigue in the general population. However, for 34% of the patients, fatigue following treatment was worse than anticipated, 39% listed fatigue as one of the three symptoms causing them most distress, 26% of patients worried about their fatigue and patients' overall quality of life was negatively related to fatigue (r = -0.46). Fatigue in disease-free patients was significantly associated with: gender, physical distress, pain rating, sleep quality, functional disability, psychological distress and depression, but not with medical (diagnosis, prognosis, co-morbidity) or treatment-related (target area, total radiation dose, fractionation) variables. The degree of fatigue, functional disability and pain before radiotherapy were the best predictors of fatigue at 9-month follow-up, explaining 30%, 3% and 4% of the variance respectively. These findings are in line with the associations found with fatigue during treatment as reported in the preceding paper in this issue. The significant associations between fatigue and both psychological and physical variables demonstrate the complex aetiology of this symptom in patients and point out the necessity of a multidisciplinary approach for its treatment.


British Journal of Cancer (1998) 78(7), 907–912
© 1998 Cancer Research Campaign

### Fatigue and radiotherapy: (B) experience in patients 9 months following treatment

**EMA Smets[1], MRM Visser[1], AFMN Willems-Groot[1], B Garssen[2], ALJ Schuster-Uitterhoeve[3] and JCJM de Haes[1]**

[1]Department of Medical Psychology J4, Academic Medical Centre, University of Amsterdam, PO Box 22700, 1100 DE Amsterdam, The Netherlands; [2]The Helen Dowling Institute for Biopsychosocial Medicine, Rotterdam; [3]Department of Radiotherapy, Academic Medical Centre, University of Amsterdam, Amsterdam, The Netherlands

**Summary** Little is known regarding the prevalence and course of fatigue in cancer patients after treatment has ended and no recurrence found. The present study examines fatigue in disease-free cancer patients after being treated with radiotherapy ($n$ = 154). The following questions are addressed. First, how do patients describe their fatigue 9 months after radiotherapy and is this different from fatigue in a non-selective sample from the general population ($n$ = 139)? Secondly, to what degree is fatigue in patients associated with sociodemographic, medical, physical and psychological factors? Finally, is it possible to predict which patients will suffer from fatigue 9 months after radiotherapy? Results indicated that fatigue in disease-free cancer patients did not differ significantly from fatigue in the general population. However, for 34% of the patients, fatigue following treatment was worse than anticipated, 39% listed fatigue as one of the three symptoms causing them most distress, 26% of patients worried about their fatigue and patients' overall quality of life was negatively related to fatigue ($r$ = –0.46). Fatigue in disease-free patients was significantly associated with: gender, physical distress, pain rating, sleep quality, functional disability, psychological distress and depression, but not with medical (diagnosis, prognosis, co-morbidity) or treatment-related (target area, total radiation dose, fractionation) variables. The degree of fatigue, functional disability and pain before radiotherapy were the best predictors of fatigue at 9-month follow-up, explaining 30%, 3% and 4% of the variance respectively. These findings are in line with the associations found with fatigue during treatment as reported in the preceding paper in this issue. The significant associations between fatigue and both psychological and physical variables demonstrate the complex aetiology of this symptom in patients and point out the necessity of a multidisciplinary approach for its treatment.

**Keywords:** fatigue, radiotherapy; psychological factor; prediction

Despite the high prevalence of fatigue in cancer patients, there is a lack of research on its causes and course over time. Studies performed are mostly restricted to the period of treatment (Smets et al, 1993). Consequently, little is known regarding the prevalence and course of fatigue following treatment. However, results from studies investigating psychological and physical distress in cancer survivors suggest that some patients continue to experience fatigue long after treatment has ended. Devlen et al (1987), for example, examined 120 newly diagnosed patients with Hodgkin's or non-Hodgkin's disease in a prospective study. Although most patients were no longer receiving treatment and were free of cancer at 1-year follow-up, 42% of these patients continued to complain of loss of energy and 32% of tiredness. Fobair et al (1986) investigated the psychological problems that developed in long-term survivors of Hodgkin's disease. At a median time since treatment of 9 years, energy had not returned to their satisfaction in 37% of the patients. In a survey among members of the Dutch patient organization of Hodgkin and non-Hodgkin patients (Breij and Visser, 1990), 61% of the subjects reported fatigue that was described as 'moderate to quite bad'. Treatment had ended more than 2 years before the survey in 60% of the sample. Lastly, Berglund et al (1991) assessed late effects of adjuvant treatment of breast cancer patients, free from recurrence 2–10 years after primary therapy. Patients who had received radiotherapy reported decreased stamina (75%) more frequently than did chemotherapy patients (61%).

*Received 17 December 1997*

*Revised 17 March 1998*

*Accepted 1 April 1998*

*Correspondence to:* EMA Smets

The mechanisms contributing to persistent fatigue in disease-free cancer patients can only be speculated upon. Permanent changes in the immune or endocrine system, resulting from treatment toxicity, might cause a person to feel more fatigued. Treatment may also have resulted in permanent changes in physical functioning such as changes in defecation pattern, in lung function caused by fibrosis of lung tissue or in hormonal functioning (Leer and Van der Schueren, 1991). These in turn may bring about symptoms such as pain or shortness of breath and impairments in daily functioning that in our study (see foregoing article) and other studies (Irvine et al, 1994; Belza, 1995) have all been found to be associated with fatigue. Immobilization has also been suggested as an explanation for persistent fatigue. Inactivity resulting from prolonged periods of bed rest reduces the capacity for activity and produces an increased sense of effort for a given level of activity (Sharpe and Bass, 1992). From a psychological perspective, it is suggested that greater fatiguability resulting from an impaired condition might induce avoidance behaviour which, in the long run, sustains feelings of fatigue (Wessely et al, 1990). Chronic fatigue is also commonly found to be related to feelings of depression or anxiety (Wessely et al, 1990; Ray, 1991; Belza, 1995).

The symptom of fatigue is not specific for cancer. Prevalence rates between 14% and 34% of tiredness have been found in community surveys (Chen. 1986: Rillsdale. 1991: Lewis and Wessely. 1992: Bensing and Schreurs. 1995). In ambulatory care. fatigue is one of the most frequently reported problems. For example. Kroenke et al (1988) reported that of the 1159 patients surveyed from primary care clinics 24% indicated that fatigue had been a major problem for a month or more. In a study involving randomly selected patients of a health care centre. 45% were scored as fatigued (Valdini et al. 1987). More recently. Fuhrer and Wessely (1995) noted in their primary care sample that about one-third of all patients reported persistent symptoms of fatigue both in a self-administered questionnaire and to their physician. Results of a primary care study in the Netherlands indicate that fatigue is the third most frequent reason reported for consulting a primary physician (van Boven and Dijksterhuizen. 1993).

To interpret the significance of results obtained in follow-up studies involving cancer patients. a comparison should therefore be made with persons without a history of cancer. Pickard-Holley (1991) made such a comparison and did not find any difference between a sample of 12 women receiving chemotherapy for ovarian cancer and a convenience sample of 12 apparently healthy women. Irvine et al (1994) compared fatigue in cancer patients ($n$ = 101) with fatigue in healthy auxiliary staff ($n$ = 53). Before the start of either radio- or chemotherapy treatment. no differences between groups were found. However. over the course of treatment. the degree of fatigue reported by patients was significantly higher than fatigue reported by the control subjects. Finally. Glaus (1993) compared the level of fatigue over 24-h periods of in-patients with cancer ($n$ = 20). in-patients with chronic inflammatory gastrointestinal disease ($n$ = 12) and healthy control subjects ($n$ = 30). The profile over the day showed significant differences between these groups. In the morning. cancer patients had the highest level of fatigue compared with the other two groups. their fatigue slowly increasing during the day. The healthy control subjects started the day without tiredness. remained fit until the late afternoon and became very fatigued in the evening. However. when the fatigue scores were averaged over a 24-h period. no significant differences between the groups were found.

These studies suffer from methodological weaknesses such as small sample sizes. heterogeneity with respect to diagnosis and treatment modality and/or the control groups being convenience samples. Also. all studies were restricted to the period of active cancer treatment.

This investigation examines fatigue in disease-free cancer patients after having been treated with radiotherapy. The research questions addressed are as follows. Firstly. how fatigued are patients 9 months after radiotherapy and how do they describe this experience. Secondly. to what degree is fatigue in disease-free patients associated with sociodemographic. medical and concurrent physical and psychological factors. Finally. is it possible to predict before the start of radiotherapy which patients will suffer from fatigue 9 months afterwards?

### METHOD

### Sample and procedure

#### Disease-free patients

Disease-free patients comprised consecutive cancer patients who had finished radiotherapy at the Academic Medical Centre in Amsterdam 9 months before and were disease free at the time of measurement. Eligibility criteria and the procedure are described in more detail in the preceding article in this issue. Patients were excluded from the 9-month follow-up when they had received additional cancer treatment following radiotherapy.

#### Reference group

The reference group consisted of a non-selective sample taken from the telephone directories of the same residential areas from which the patients were derived. A letter was sent to the selected residences to introduce the study. which was followed by a telephone call by the researchers. In order to prevent an overrepresentation of women. on the grounds of being more frequently at home when approached for participation. the next person to have a birthday within that residence was asked to participate. Respondents were to be at least 18 years of age.

Out of practical considerations. most respondents were requested to complete a home-sent questionnaire. To investigate a possible bias introduced by the difference in method as compared with the patients. a subgroup of respondents was invited to be interviewed at their home.

Respondents who declined participation were asked to give their date of birth and to rate their fatigue on a scale from 0 to 10 in order to be able to assess selective drop-out.

### Instruments

#### Disease-free patients

All standard instruments used in the disease-free patients were similar to those described in the preceding article. Also. the same information from their medical charts was used (diagnosis. prognosis. radiation area and dose. fractionation). The following additional data were collected on interview: co-morbidity. the course of fatigue since end of treatment. frequency of fatigue (never. hardly ever. sometimes. most of the time or always). the time of most intense fatigue during the day (no clear pattern. early morning. noon. afternoon. late afternoon. evening). physical sensations associated with fatigue (muscle weakness. sweating. uncomfortable feeling in the chest. blurred sight and shortness of breath: with response categories not at all. a bit. moderate and very much). hours of sleep. the degree of concern (not at all. a bit. moderate. very much) and a comparison of their current fatigue with fatigue during the previous month (less intense. no difference. more intense) and with their expectations (worse than anticipated. as expected. better than anticipated).

#### Reference group

Sociodemographic characteristics were recorded. Respondents completed the Multidimensional Fatigue Inventory (MFI-20) and the numerical scale for the assessment of fatigue. As in patients. additional questions addressed the frequency of fatigue. the time of most intense fatigue during the day. physical sensations associated with fatigue. hours of sleep and perceived cause of fatigue.

### Statistical methods

Analyses involved descriptive statistics and one-way analyses of variance (ANOVAs) for the description of fatigue. To establish a possible effect of method of assessment in the reference group. MFI scores for the interview and questionnaire groups were compared using analyses of variance. As before. the score for

© Cancer Research Campaign 1998

**Table 1** Sample characteristics of disease-free patients ($n$ = 154) and normal controls ($n$ = 139)

| | Patients | | Controls | |
|---|---|---|---|---|
| | n | % | n | % |
| Mean age (years) | 65 ± 12 | | 46 ± 16[a] | |
| Gender | | | | |
| Female | 66 | 43 | 78 | 56[a] |
| Male | 88 | 57 | 61 | 44 |
| Education level | | | | |
| Less than high school | 37 | 24 | 11 | 8 |
| Lower educational level | 48 | 31 | 34 | 24 |
| High school | 43 | 28 | 57 | 41 |
| Advanced graduate degrees | 26 | 17 | 35 | 25 |
| Marital status | | | | |
| Married | 111 | 72 | 73 | 53 |
| Living together | 6 | 4 | 15 | 11 |
| Single | 19 | 12 | 39 | 28 |
| Widowed | 18 | 12 | 10 | 7 |
| (Co)-morbidity | 74[b] | 52 | 43 | 31 |
| No. of patients with cancer diagnosis | | | | |
| Head and neck | 8 | 5 | | |
| Gastrointestinal | 7 | 5 | | |
| Gynaecological | 19 | 12 | | |
| Lung | 11 | 7 | | |
| Breast | 30 | 20 | | |
| Prostate | 48 | 31 | | |
| Testis | 7 | 5 | | |
| Other genitourinary tract | 7 | 5 | | |
| Haematological malignancies | 10 | 7 | | |
| Miscellaneous | 7 | 3 | | |

[a]Difference between patients and reference group, $P < 0.0001$. [b]Co-morbidity was unknown for ten patients.

general fatigue of the MFI was used as dependent variable and general fatigue will be referred to as 'fatigue'. Pearson product moment correlations and ANOVAs were used to assess bivariate, concurrent associations. Stepwise regression analyses were used for the multivariate prediction of general fatigue at follow-up, using data from the pretreatment assessment as predictors. The same grouping procedure was followed as described previously, as were other analysis procedures concerning interactions, overlap in item content and grouping on the basis of radiation target area.

### RESULTS

### Sample

### *Disease-free patients*

Of the original 250 participating patients, at the time of follow-up, 18 (7%) had died, 42 (17%) were excluded because of additional cancer treatment during or following radiotherapy, eight (4%) could not be interviewed for logistic reasons, 13 (5%) declined further participation and 15 (6%) had active disease. Sociodemographic and clinical characteristics of the remaining 154 (62%) patients without apparent disease are presented in Table 1.

### *Reference group*

Of the 123 persons approached to complete the questionnaire, 106 (86%) initially agreed. Thirteen persons (12%) failed to comply, resulting in a 'questionnaire' sample of 93 persons (74%). Of the 81 persons requested for an interview, 48 (59%) agreed to participate. Two persons were subsequently excluded because they had a history of cancer, resulting in an 'interview' sample of 46 persons.

Comparison of the fatigue scores on the MFI of the interview and questionnaire groups yielded no differences. The two groups were therefore combined. Sociodemographic characteristics of the final sample ($n$ = 139) are included in Table 1.

Those respondents who refused participation were found to be older (58 vs 46 years; $F(1,166) = 14.11$, $P < 0.0005$) and more fatigued (mean 5.5 ± 2.3 vs 3.7 ± 2.7; $F(1,163) = 12.42$, $P < 0.001$) than participants. No difference was found with regard to gender distribution.

### *Comparison of patients and reference group*

The patient group contained more men ($Z = -2.149$, $P < 0.01$) and was older [$F(1,287) = 129.8$, $P < 0.0001$)] than the reference group. When controlling for age and gender distribution, no differences between the samples were found with respect to level of education or prevalence of co-morbidity.

### Course, dimensions and intensity of fatigue

In Table 2, the mean scores for the five dimensions of fatigue are presented for the patients at follow-up and the reference group. When controlling for age and gender, no differences in numerical fatigue scores and in general fatigue, physical fatigue, reduced activity and reduced motivation were found between the two samples. A trend emerged for mental fatigue, with the reference group reporting more difficulties in cognitive functioning [$F(1,281) = 2.96$, $P = 0.08$].

Table 2 Mean scores for the five fatigue scales for disease-free patients and general population (range 4–20)

| | Disease-free patients ($n$ = 154) | | General population ($n$ = 139) | |
|---|---|---|---|---|
| | Mean | s.d. | Mean | s.d. |
| General fatigue | 10.15 | 5.2 | 9.91 | 5.2 |
| Physical fatigue | 9.77 | 5.0 | 8.79 | 4.9 |
| Reduced activity | 9.67 | 4.7 | 8.69 | 4.6 |
| Reduced motivation | 8.18 | 4.6 | 8.23 | 4.0 |
| Mental fatigue | 6.95 | 4.2 | 8.33 | 4.8 |

Table 3 Bivariate associations with general fatigue for disease-free patients ($n$ = 154) 9 months after radiotherapy

| Domains and their variables | Statistic | P |
|---|---|---|
| Sociodemographical | | |
| Sex | $F(1.150) = 8.08$ | <0.01 |
| Age | $r = -0.01$ | NS |
| Education | $F(3.148) = 2.11$ | NS |
| Medical | | |
| Diagnosis | $F(6.139) = 1.50$ | NS |
| Prognosis | $r = -0.07$ | NS |
| Co-morbidity | $F(1.140) = 3.43$ | 0.07 |
| Radiotherapy | | |
| Dose | −0.18. 0.16. −0.17[a] | NS |
| Fractionation | −0.07. 0.07. −0.13[a] | NS |
| Physical | | |
| Physical distress | $r = 0.51$ | <0.001 |
| Pain | $r = 0.40$ | <0.005 |
| Quality of sleep | $r = 0.26$ | <0.001 |
| Hours of sleep | $r = 0.24$ | <0.001 |
| Day-time napping | $F(1.150) = 17.87$ | <0.0001 |
| Performance status | $r = 0.55$ | <0.001 |
| Psychological | | |
| Psychological distress | $r = 0.36$ | <0.001 |
| Depression | $r = 0.49$ | <0.001 |
| Optimism | $r = -0.10$ | NS |
| Neuroticism | $r = 0.11$ | NS |

[a]For patients radiated on the head and neck. thorax and abdomen/pelvis respectively.

### Characteristics of the fatigue experience

### *Frequency and intensity*

About half of the patients recalled having been fatigued during the first 3 months following radiotherapy (32% moderate and 19% very much). whereas the remaining patients reported a bit (19%) or no fatigue (30%). For 52% of the patients fatigue subsequently decreased until it remained stable or completely disappeared. 13 patients (10%) reported a gradual increase in fatigue and another eight patients (5%) reported a return of their fatigue after an initial decrease.

Fatigue after radiotherapy was not as bad as expected for 49% of the patients: 34% reported it to be worse than anticipated. For 39% of patients. fatigue at follow-up was reported as one of the three symptoms that caused them most distress and 26% expressed some concern regarding their fatigue. Fatigue correlated −0.45 ($P < 0.001$) with the patients' overall quality of life.

The following percentages are restricted to the patients who reported a fatigue score of greater than 1 on the numerical rating scale [90 patients (58%) and 103 respondents from the reference group (74%)].

### *Time pattern*

Seventy-four per cent of the patients reported no difference between their current fatigue and fatigue in the previous month. 10% less intense fatigue and 16% more intense fatigue. In the reference group. these percentages were 50%. 16% and 34% respectively. Fatigue was generally most intense in the evening for 24% of the patients: 39% could not identify a clear pattern. Of the reference group. 22% was most fatigued in the late afternoon. 28% in the evening and 14% experienced no clear pattern.

### *Associated symptoms*

The symptoms most frequently associated with fatigue were sweating (28%) and shortness of breath (24%) in patients. and sore muscles (37%) and blurred sight (28%) in the reference group.

### *Rest*

When controlling for age. no significant differences between the two samples in frequency and duration of day-time napping. nor in amount of night-time sleep appeared.

### Associations with fatigue at follow-up

Results of the analyses regarding the concurrent. bivariate associations between fatigue and other factors at 9-month follow-up are presented in Table 3. Women reported more fatigue than men. All physical variables were associated with fatigue. indicating more fatigue with a higher degree of physical distress. including pain. and functional disability. and a more impaired quality of sleep. Patients slept more with increased levels of fatigue. both during the day and the night. Fatigue also increased with increasing levels of psychological distress. No associations were found with medical or treatment-related variables.

### Prediction of fatigue 9 months after treatment

Results of the regression analyses are presented in Table 4. Of the sociodemographic characteristics (age. gender. education). only gender explained 5% of the variance in fatigue of patients at follow-up. None of the medical (diagnosis. prognosis. co-morbidity) or treatment-related (total radiation dose and fractionation) variables predicted fatigue at follow-up. Regarding the domain of physical predictors of fatigue (physical distress. pain. quality of sleep. fatigue and functional disability. all measured pretreatment) the same interactions as discussed in the preceding paper were considered for inclusion in the regression analysis. but again there was no evidence supporting the hypotheses. and therefore interaction terms were not included. Pretreatment fatigue. the degree of functional disability and pain at that time explained 29%. 3% and 2% of the variance in fatigue at follow-up respectively. The same analysis. excluding pretreatment fatigue. resulted in 22% of the variance in fatigue being explained by the degree of pretreatment functional disability only.

Regarding the psychological domain (neuroticism. optimism. psychological distress and depression). the analysis (without interactions) showed pretreatment psychological distress to explain 8% of the variance in fatigue at follow-up.

© Cancer Research Campaign 1998

**Table 4** Significant pretreatment predictors of general fatigue scores at follow-up, using stepwise regression analyses

| Domain | Predictor | *R* | $R^2$ | Regression coefficient | | |
|---|---|---|---|---|---|---|
| | | | | B | s.e. B | *P* |
| 1 Sociodemographical | Gender | 0.23 | 0.05 | 2.38 | 0.84 | <0.01 |
| 2 Physical | Pretreatment fatigue | 0.54 | 0.29 | 0.42 | 0.09 | <0.0001 |
| | Functional disability | 0.03 | 0.03 | 0.14 | 0.06 | <0.05 |
| | Pain | 0.02 | 0.02 | −1.89 | 0.89 | < 0.05 |
| 2a Physical, not including pretreatment fatigue | Functional disability | 0.45 | 0.20 | 0.26 | 0.05 | <0.0001 |
| 3 Psychological | Psychological distress | 0.27 | 0.08 | 0.39 | 0.12 | <0.005 |
| Combined (1,2,3) | Pretreatment fatigue | 0.55 | 0.30 | 0.41 | 0.09 | <0.0001 |
| | Functional disability | 0.03 | 0.03 | 0.15 | 0.05 | <0.01 |
| | Pain | 0.02 | 0.04 | −2.08 | 0.86 | <0.05 |
| Combined, not including pretreatment fatigue (1,2a,3) | Functional disability | 0.47 | 0.22 | 0.26 | 0.05 | <0.0001 |

A subsequent overall regression analysis included the four variables that significantly predicted fatigue within their separate domains, taking their interrelatedness into account (see Table 4). The degree of fatigue, functional disability and pain at pretreatment contributed to the prediction of fatigue at follow-up, explaining 30%, 4% and 3% of the variance respectively. When pretreatment fatigue was not included, 22% of the variance in outcome was explained by the degree of post-treatment functional disability.

### DISCUSSION

This is, to the best of our knowledge, the first investigation that has set out to investigate chronic fatigue in disease-free cancer patients. The only related study was conducted by Bloom et al (1990), who investigated energy expenditure in patients with Hodgkin's disease 1–5 years after treatment.

The lack of difference in fatigue scores between disease-free patients and the reference group is noteworthy because the former group was expected to be more fatigued. Despite some reservations, which will be discussed later, this finding challenges the implicit assumption in studies on long-term effects that complaints of fatigue or lack of energy are characteristic for cancer survivors. As described, high prevalence rates of complaints of chronic fatigue are found in general population and primary care studies as well. In addition, previous investigations that included a non-cancer comparison group have also demonstrated comparable fatigue ratings for cancer patients and control subjects, before (Irvine et al, 1994) or during treatment (Pickard-Holley 1991; Glaus, 1993).

However, examination of the description of the fatigue experience does indicate some interesting differences between the two samples, which show nuances in the apparent equivalence in fatigue. More patients than respondents from the reference group (74% vs 55%) reported their fatigue to have been stable during the month before assessment, suggesting fatigue to be a more chronic condition in patients. Fatigue in patients also appears to be more unpredictable, as indicated by the finding that 39% of patients experienced no clear pattern in the onset of their fatigue compared with 14% of the reference group. The symptoms mostly associated with fatigue also differ between the two samples: sweating and shortness of breath in patients, painful muscles and bad sight in the reference group. These findings suggest that maybe not the intensity but the characteristics of fatigue in cancer patients are different from what is found in the general population. Glaus (1996) reached a similar conclusion on the basis of a qualitative comparison of the description of fatigue by cancer patients and healthy control subjects.

Another reservation with respect to the finding of equivalent fatigue scores in patients and the reference group is the problem of 'response shift'. The term response shift refers to the change in a person's internal standard for determining his or her level of functioning on a given dimension (Breetvelt en van Dam, 1991; Sprangers, 1996). The experience of fatigue during radiotherapy could have changed a patient's standard of measurement concerning fatigue. What has been perceived to be 'intense' fatigue before treatment, might be labelled 'slightly' fatigued after having experienced exhaustion during treatment. The possible occurrence of a response shift complicates the interpretation of comparison data.

Finally, patients may limit their activities to such a degree that, as a result, their fatigue does not exceed the level found in the general population.

Our findings suggest that medical characteristics such as diagnosis, prognosis and co-morbidity, and treatment characteristics such as total radiation dose, target area and fractionation are unrelated to long-term fatigue. As indicated in the preceding article, this lack of impact may result from the heterogeneity of the study population and the crude assessment categories used.

The association between fatigue and psychological distress found, both concurrently and prospectively, is consistent with the results from other research both in cancer (Nerenz et al, 1982; Fobair et al, 1986; Jamar, 1989; Blesh, 1991) and non-cancer populations (Fisk et al, 1994; Fuhrer and Wessely, 1995; Belza, 1995). It underlines that the role of psychological distress should be taken into account when trying to alleviate fatigue.

As in the treatment-related study, no association between fatigue and neuroticism was found. This was unexpected, because negative affectivity has been found to correlate consistently and moderately with various measures of health complaints and physical symptoms (Watson and Pennebaker, 1989). This finding suggests that fatigue reported by these patients cannot be considered to reflect a general tendency to complain. The lack of an association with optimism is more in line with the conclusion from Watson (1988) that positive effect measures are generally found to be unrelated to self-reported health problems.

© Cancer Research Campaign 1998

Functional disability, fatigue and pain, assessed before the start of treatment, together explained 37% of the variance in fatigue at follow-up. This is a considerable amount of variance explained when taking into account that the variance in one symptom, more than 9 months later is predicted. However, it also demonstrates that it remains difficult to predict, on an individual basis, who will suffer from long-term fatigue.

The findings presented must be considered within the limitations of this study. Our reference group was not necessarily an unbiased sample of the Dutch general population. Respondents were approached by means of telephone directories, which does not cover individuals who lack a telephone or those with unlisted numbers. Subjects who are registered tend to be more educated than subjects who are not (Brambilla and McKinlay, 1987). Also, by being dependent on telephone contact, persons being more frequently at home might be overrepresented. In view of the low response rate in the interview sample, other selection processes may also have affected the representativeness of this sample. Respondents who refused to participate were found to be significantly more fatigued than participants. As previously stated, patients who refused study participation were also found to be more tired than participants. As a result, the degree of fatigue of both patients and the general population in this investigation might be an underestimation of the problem.

Notwithstanding these limitations, the significant associations found in this investigation between fatigue and both psychological and physical variables again highlights that fatigue is a symptom with multiple factors contributing to its manifestation. Consequently, a multidisciplinary approach seems warranted both for its investigation and treatment. Physical therapists in particular may offer a valuable contribution given the prominent role of functional disability in the prediction of fatigue.

Finally, one must beware of concluding that fatigue is a trivial complaint. Concluding that cancer patients 9 months after radiotherapy seem not to differ from a population sample does not imply that fatigue is clinically irrelevant. More than a third of the patients listed fatigue as one of the three symptoms causing them most distress, it worried about a quarter of the patients and it was negatively and substantially related to the patient's evaluation of their quality of life. These data indicate that fatigue can be a very disturbing complaint.

### REFERENCES

Belza BL (1995) Comparison of self-reported fatigue in rheumatoid arthritis and controls. *J Rheumatol* **22**: 639–643

Bensing JM and Schreurs K (1995) Sexeverschillen bij moeheid (Gender differences in fatigue). *Huisarts en Wetenschap* **38**: 412–421

Berglund G, Bolund C, Fornander T, Rutqvist LE and Sjöden P-O (1991) Late effects of adjuvant chemotherapy and postoperative radiotherapy on quality of life among breast cancer patients. *Eur J Cancer* **27**: 1075–1081

Blesh K, Paice JA, Wickham R and Harten N, Schnoor DK, Purl S, Rehwalt M, Lamm Kopp P, Manson S, Barry Coveny S, McHale M and Cahill M (1991) Correlates of fatigue in people with breast or lung cancer. *Oncol Nurs Forum* **18**: 81–87

Bloom JR, Gorsky RD, Fobair P, Hoppe R, Cox RS, Varghese A and Spiegel D (1990) Physical performance at work and at leisure: validation of a measure of biological energy in survivors of Hodgkin's disease. *J Psychsoc Oncol* **8**: 49–63

Boven van K and Dijksterhuizen P (1993). *De schatbare waarde van aanvullend onderzoek in de huisartspraktijk*. Thesis, University of Amsterdam, Meditekst Lelystad

Brambilla DJ and McKinlay SM (1987) A comparison of responses to mailed questionnaires and telephone interviews in a mixed mode health survey. *Am J Epidemiol* **126**: 962–971

Breetvelt IS and Dam FSAM van (1991) Under reporting by cancer patients: the case of response-shift. *Soc Sci Med* **32**: 981–987

Breij GCC and Visser-Wolf PJ De (1990) *Investigation Hodgkin-Contactgroup*. Research report, University of Utrecht

Chen MK (1986) The epidemiology of self-perceived fatigue among adults. *Preventive Med* **15**: 74–81

Devlen J, Maguire P, Philips P, Crowther D and Chambers H (1987) Psychological problems associated with diagnosis and treatment of lymphomas. 1. retrospective; 2. prospective. *Br Med J* **295**: 953–957

Fisk JD, Pontefract A, Ritvo PG, Archibald CJ and Murray TJ (1994) The impact of fatigue on patients with multiple sclerosis. *Can J Neurol Sci* **21**: 9–14

Fobair P, Hoppe RT, Bloom J, Cox R, Vaughese A and Spiegel D (1986) Psychosocial problems among survivors of Hodgkin's disease. *J Clin Oncol* **4**: 805

Fuhrer R and Wessely S (1995) The epidemiology of fatigue and depression: a French primary-care study. *Psychol Med* **25**: 895–905

Glaus A (1993) Assessment of fatigue in cancer and non-cancer patients and in healthy individuals. *Support Care Cancer* **1**: 305–315

Glaus A, Crow R and Hammond S (1996) A qualitative study to explore the concept of fatigue/tiredness in cancer patients and in healthy individuals. *Support Care Cancer* **4**: 82–96

Irvine D, Vincent L, Graydon JE, Bubela N and Thompson L (1994) The prevalence and correlates of fatigue in patients receiving treatment with chemotherapy and radiotherapy. *Cancer Nursing* **5**: 367–378

Jamar S (1989) Fatigue in women receiving chemotherapy for ovarian cancer. In *Key Aspects of Comfort, Management of Pain Fatigue and Nausea*, Funk SG, Tornquist EM, Campagne MT, Archer Gopp L and Wiese RA (eds), pp. 224–228. Springer: New York

Kroenke K, Wood DR, Mangelsdorff AD, Meier N and Powell JB (1988) Chronic fatigue in primary care. Prevalence, patient characteristics and outcome. *JAMA* **260**: 929–934

Leer JWH and Schueren Van Der E (1996) Radiotherapeutic principles. In *Oncology*, van de Velde CJH, Bosman FT and Wagener DJTh (eds), Bohn Stafleu van Loghum, Houten/Diegem

Lewis G and Wessely S (1992). The epidemiology of fatigue: more questions than answers. *J Epidemiol Comm Health* **46**: 92–97

Nerenz DR, Leventhal H and Love RR (1982) Factors contributing to emotional distress during cancer chemotherapy. *Cancer* **50**: 1020–1027

Pickard-Holley S (1991) Fatigue in cancer patients. A descriptive study. *Cancer Nursing* **14**: 13–19

Ray C (1991) Editorial: Chronic fatigue syndrome and depression: conceptual and methodological ambiguities. *Psychol Med* **21**: 1–9

Rillsdale L (1991) Tired all the time. *Br Med J* **303**: 1490–1491

Sharpe M and Bass C (1992) Pathophysiological mechanisms in somatization. *Int Rev Psychiatr* **4**: 81–97

Smets EMA, Garssen B, Schuster-Uitterhoeve ALJ and De Haes JCJM (1993) Fatigue in cancer patients. *Br J Cancer* **68**: 220–224

Sprangers MAG (1996) Response-shift bias: a challenge to the assessment of patients quality of life in cancer clinical trials. *Cancer Treat Rev* **22**: 55–62

Valdini AF, Steinhardt SI and Jaffe AS (1987) Demographic correlates of fatigue in a university family health centre. *Family Practise* **4**: 103–107

Watson D and Pennebaker JW (1989) Health complaints, stress and distress. Exploring the central role of negative affectivity. *Psychol Rev* **96**: 234–254

Watson D, Clark LA and Tellegen A (1988) Development and validation of brief measures of positive and negative affect: the PANAS scales. *J Pers Soc Psych* **54**: 1063–1070

Wessely S, Butler S, Chalder T and David A (1990) The cognitive behavioural management of the postviral fatigue syndrome. In *The Postviral Fatigue Syndrome*. Jenkins R and Mowbray J (eds), pp. 305–334. Wiley: Chichester

© Cancer Research Campaign 1998